# Ernährung, Stoffwechsel, Gehirn und mentale Gesundheit

**DOI:** 10.1007/s00115-024-01678-6

**Published:** 2024-06-17

**Authors:** Denise Linsmayer, Gunter P. Eckert, Julia Reiff, Dieter F. Braus

**Affiliations:** 1 Vitos Klinikum Rheingau, Kloster-Eberbach-Straße 4, 65346 Eltville, Deutschland; 2 Institut für Ernährungswissenschaft, Justus-Liebig-Universität Gießen, Wilhelmstraße 20, 35392 Gießen, Deutschland

**Keywords:** Adipositas, Dopamin, Diabetes, Insulinrezeptoren, Neurodegeneration, Obesity, Dopamine, Diabetes, Insulin receptors, Neurodegeneration

## Abstract

Der Beitrag untersucht den komplexen Zusammenhang zwischen Ernährung, Stoffwechsel, Gehirnfunktion und mentaler Gesundheit. Er beleuchtet zwei zentrale, sich ergänzende Modelle – das Energiebilanzmodell und das Kohlenhydrat-Insulin-Modell –, um die Entstehung von Adipositas und metabolischen Dysfunktionen zu verstehen. Besondere Aufmerksamkeit wird zum einen der Rolle von Dopamin in der Ernährungsregulation und zum anderen von Insulin im Gehirn gewidmet, welche beide wesentlich an der Pathogenese neurodegenerativer und stressassoziierter psychischer Störungen beteiligt sind. Zudem wird die Bedeutung des Schlafes, der Ernährungsgewohnheiten wie beispielsweise medizinisch begleitete Kalorienrestriktion für mentale Gesundheit und das Konzept des „Brain Food“ beschrieben. Die Befunde verdeutlichen die Relevanz der Ernährungsmedizin für die Psychiatrie und Psychotherapie und der Berücksichtigung von Stoffwechselzuständen für die Prävention und Behandlung psychischer und neurodegenerativer Erkrankungen.

## Abstract (en)

This review article explores the intricate relationship between nutrition, metabolism, brain function and mental health. It highlights two key complementary models: the energy balance model and the more comprehensive carbohydrate-insulin model, to understand the development of obesity and metabolic dysfunctions. It particularly focuses on the role of dopamine in dietary regulation and insulin in the brain, both of which are crucial in the pathogenesis of neurodegenerative and stress-associated mental disorders. Additionally, the significance of sleep and dietary habits, such as medically assisted calorie restriction for mental health and the concept of “brain food” are described. These findings emphasize the importance of nutritional medicine in psychiatry and psychotherapy and the consideration of metabolic states for the prevention and treatment of mental and neurodegenerative diseases.

## Lernziele

Nach der Lektüre dieses Beitrags …

- verstehen Sie die Grundlagen des Energiebilanz- und des speziellen Aspekts des Kohlenhydrat-Insulin-Modells,
- erkennen Sie den Zusammenhang zwischen Adipositas, Ernährung und Neurodegeneration,
- haben Sie einen Einblick in die Rolle der Insulinrezeptoren im Gehirn und deren Auswirkungen auf die kognitive Funktion,
- verstehen Sie besser die Verbindung zwischen Schlaf, Ernährung und mentaler Gesundheit,
- können Sie den Nutzen von „Brain Food“ zur Vorbeugung psychischer Störungen genauer einordnen.

## Hintergrund

Die weltweit zunehmende Prävalenz von **Stoffwechselstörungen**

Stoffwechselstörungen

wie Adipositas und Diabetes mellitus Typ 2 (T2DM) stellt nicht nur eine Herausforderung für das Gesundheitssystem dar, sondern beeinflusst auch erheblich die **psychische Gesundheit**

psychische Gesundheit.

Aktuelle Studien verdeutlichen einen engen Zusammenhang zwischen gesünderer und bewussterer Lebensweise, Ernährungsgewohnheiten, metabolischen Störungen und der Entstehung sowie dem Verlauf vieler chronischer Erkrankungen und eben auch psychischer Störungen.

Neben einer Vielzahl von Inkretinen, Botenstoffen und im Gehirn aktiven Signalwegen wie z. B. mTOR („mammalian target of rapamycin“) rückt auch die Wirkung von **Insulin**

Insulin,

welche sowohl den Glukosestoffwechsel als auch kognitive Funktionen und neuronale Netzwerke beeinflusst, in den Fokus. Dies hat zu einem gesteigerten Interesse an der Rolle spezifischer Nährstoffe, dem sogenannten **„Brain Food“**

„Brain Food“,

für die mentale Gesundheit geführt, ebenso wie für medizinisch begleitetes Fasten als potente Präventionsmaßnahme. Auch der Schlaf spielt eine wichtige Rolle in diesem Zusammenhang.

Dieser Artikel präsentiert eine Auswahl aktueller Befunde zu diesen Themen für den praktischen Alltag und diskutiert deren Einfluss auf die Prävention und Behandlung von Stoffwechsel- und psychischen Erkrankungen, mit dem Ziel, Empfehlungen für die klinische Praxis in der Psychiatrie und Psychotherapie abzuleiten.

## Wie kommt es zu Übergewicht und Adipositas?

Adipositas, T2DM und das metabolische Syndrom (MetS) haben eine **komplexe Genese**

komplexe Genese,

resultieren aber im Wesentlichen aus einer polygenetischen Vulnerabilität und einer Über- bzw. Fehlernährung in Verbindung mit mangelnder körperlicher Aktivität. Übergewicht (Body-Mass-Index [BMI] 25–30 kg/m^2^) und Adipositas (BMI > 30 kg/m^2^) stellen sowohl in Deutschland [1] als auch weltweit relevante **gesundheitliche Risikofaktoren**

gesundheitliche Risikofaktoren

sowohl somatisch als auch psychisch dar [2].

Die **konventionelle Behandlung**

konventionelle Behandlung

von Fettleibigkeit, die auf dem Ersten Hauptsatz der Thermodynamik basiert, geht davon aus, dass überschüssige Körperfettzunahme durch übermäßiges Essen angetrieben wird und dass alle Kalorien in dieser Hinsicht metabolisch gleich sind. Um Gewicht zu verlieren, muss man also letztendlich weniger essen und sich mehr bewegen. Dieses Rezept ist jedoch bei vielen Menschen auf lange Sicht nicht erfolgreich, einerseits, weil unbegleitete vollständige **Kalorienrestriktion**

Kalorienrestriktion

vorhersehbare biologische Reaktionen auch im Gehirn hervorrufen kann, die einer kontinuierlichen Gewichtsabnahme entgegenstehen. Andererseits gibt es auch biologische Wechselwirkungen zwischen der Lebensmittelverarbeitung, dem Mikrobiom, dem Stoffwechselaufwand des Wirts, dem Ernährungsverhalten und dem Gehirn sowie auch intergenerationeller Übertragung von Risiken.

Das **Kohlenhydrat-Insulin-Modell**

Kohlenhydrat-Insulin-Modell

[3], welches in der Wissenschaft als mögliche Spezifizierung des beschriebenen Energiebilanzmodells eingeordnet wird [4], jedoch in der Stoffwechselforschung nicht unumstritten ist [4, 5], betont stärker die adaptive **Rolle des Gehirns**

**Abb. 1:**
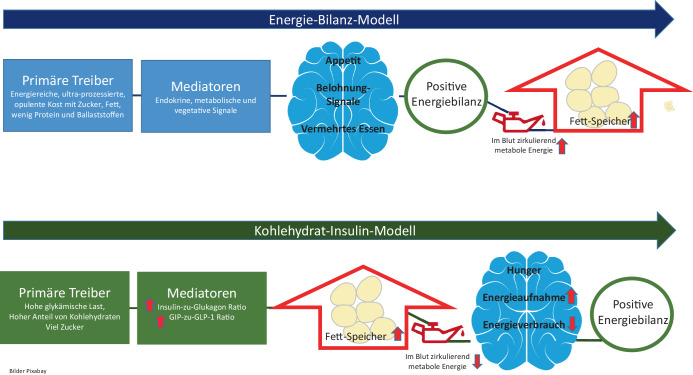
Die Konzeptualisierung von Fettleibigkeit als Störung des Energiehaushalts stellt ein physikalisches Prinzip dar, ohne die biologischen Mechanismen zu berücksichtigen, welche die Gewichtszunahme begünstigen. Ein ergänzendes Paradigma, das Kohlenhydrat-Insulin-Modell (*CIM*), modifiziert die kausale Richtung. Laut CIM führt die Erhöhung der Fettablagerung im Körper, die sich u. a. aus den hormonellen Reaktionen auf eine Ernährung mit hoher glykämischer Last ergibt, zu einer positiven Energiebilanz. *GIP* glukoseabhängiges insulinotropes Polypeptid, *GLP* „glucagon-like peptide 1“. (Mod. nach [7])

Rolle des Gehirns:

Übermäßiges Essen führt nicht allein zu einer Zunahme des Körperfetts, sondern auch der Prozess der Speicherung von überschüssigem Fett führt zu übermäßigem Essen. Eine Ernährung, die reich an hochverdichteten Kohlenhydraten ist, erhöht das Verhältnis von Insulin zu Glukagon und verlagert die Energieverteilung in Richtung Speicherung im Fettgewebe, sodass weniger Kalorien für stoffwechselaktive und kraftstoffempfindliche Gewebe im Körper übrigbleiben. Infolgedessen steigt über neuronale Prozesse im Gehirn der Hunger und die Stoffwechselrate verlangsamt sich bei dem Versuch des Körpers, Energie zu sparen. Eine kleine Verschiebung in der Substratverteilung wäre dabei für die langsame, aber fortschreitende Gewichtszunahme verantwortlich, die für häufige Formen der Fettleibigkeit charakteristisch ist (Abb. 1; [3]). Aus dieser Perspektive läuft die herkömmliche kalorienreduzierte, fettarme Diät auf eine symptomatische Behandlung hinaus, die bei einem Teil der Betroffenen nicht auf die zugrunde liegende Veranlagung zu übermäßiger Fettablagerung abzielt [6]. Eine diätetische Strategie zur **Senkung der Insulinsekretion**

Senkung der Insulinsekretion

könnte dagegen die Wirksamkeit des langfristigen Gewichtsmanagements und der Vorbeugung chronischer Krankheiten erhöhen.

Es konnte z. B. gezeigt werden, dass eine konsequent **kohlenhydratarme Ernährung**

kohlenhydratarme Ernährung,

reich an Fettsäuren, eine **insulinresistente Dyslipoproteinämie**

insulinresistente Dyslipoproteinämie

ohne negative Auswirkungen auf das LDL(Low-density-Lipoprotein)-Cholesterin verbesserte [8]. In Übereinstimmung mit dem Kohlenhydrat-Insulin-Modell erhöhte diese Senkung der verdichteten Kohlenhydrate in der Nahrung wieder den Energieverbrauch (und den Grundumsatz) während der Aufrechterhaltung der Gewichtsabnahme. Dieser metabolische Effekt kann den Erfolg der Behandlung von Fettleibigkeit verbessern, insbesondere bei Personen mit hoher Insulinsekretion [9]. Wie jedoch so häufig scheinen dabei extreme Low-carb-Ernährungsformen und Diäten eher ungünstig und mit einer erhöhten Mortalität einherzugehen [10]. Außerdem ist hochverarbeitete Nahrung unabhängig von Makronährstoffzusammensetzung adipogen.

Neben Insulin sind verschiedene **Inkretine**

Inkretine

wie Glucagon-like-Peptid‑1 (GLP-1) und glukoseabhängiges insulinotropes Polypeptid (GIP) im Darm in die Glukosehomöostase sowie die Appetitregulation involviert und stellen möglicherweise weitere Targets in der Behandlung der Adipositas dar [11].

Metformin und GLP-1-Agonisten können den Effekt zusätzlich pharmakologisch unterstützen (s. unten).

### Merke

Das Kohlenhydrat-Insulin-Modell postuliert Adipositas als Stoffwechselstörung mit hormonellen Reaktionen auf Veränderungen der Nahrungsmittel, insbesondere hochverarbeitete mit hoher glykämischer Belastung. Es ist in der Stoffwechselforschung nicht unumstritten.

## Ernährung und Psyche

Der positive Einfluss einer pflanzenbetonten, wenig verarbeiteten und **vollwertigen Ernährung**

vollwertigen Ernährung

auf unsere **geistige Verfassung**

geistige Verfassung

und Kognition ist nicht neu, jedoch liegen bislang wenige Daten dazu vor, welche Ernährungsmuster besonders empfehlenswert sind und wodurch sich diese genau positiv auswirken.

**Mikronährstoffe**

Mikronährstoffe

wie Polyphenole, Omega-3-Fettsäuren, einzelne Vitamine sowie Ballaststoffe und deren präbiotische Aktivität, aber auch antientzündliche diätetische Maßnahmen beeinflussen wohl Biomarker und molekulare Mechanismen, die bei **psychischen Erkrankungen**

psychischen Erkrankungen,

insbesondere affektiven Störungen, eine Rolle spielen. Obschon nicht alles geklärt ist, sind wohl Ernährungsinterventionen perspektivisch wichtige Ergänzungen zu herkömmlichen psychiatrischen Therapien und werden Einzug in die klinische Behandlung psychischer Störungen halten (sog. **„nutritional psychiatry“**

„nutritional psychiatry“;

[12]). Aber auch unter präventivem Aspekt und der Erhöhung von Selbstwirksamkeit sind verschiedene Modifikationen der Ernährungsweisen interessant, nicht nur bei Angsterkrankungen und Depressionen [13, 14].

Auch **psychotische Störungen**

psychotische Störungen

gehen oft mit einer unausgewogenen Ernährung einher, charakterisiert durch einen hohen Verzehr von **raffinierten Kohlenhydraten**

raffinierten Kohlenhydraten

und Fetten z. B. in Fertigprodukten und Süßgetränken, und einem niedrigen Konsum an Ballaststoffen, Omega-3- und Omega-6-Fettsäuren, Vitaminen und Mineralien. Es wurden überdies Zusammenhänge zwischen **Lebensmittelallergien**

Lebensmittelallergien

bzw. -empfindlichkeiten und der Zusammensetzung des **Mikrobioms**

Mikrobioms

(siehe Infobox 1) bei an einer psychotischen Störung Leidenden festgestellt. Es ist jedoch nicht eindeutig geklärt, ob spezifische Ernährungsmuster an der Entstehung bzw. Aufrechterhaltung dieser beteiligt sind oder ob Menschen mit bestehenden psychotischen Störungen zu schlechteren Essgewohnheiten neigen [15].

Neuere Daten fanden einen Einfluss von **Insulin**

Insulin

auf zentrale Neurotransmittersysteme. Insulinresistenz und Hypoinsulinämie (s. unten) scheinen vor allem das glutamaterge sowie das **dopaminerge System**

dopaminerge System

zu beeinflussen mit Beeinträchtigungen der synaptischen Plastizität und der Funktion von Glutamatrezeptoren. Zusätzlich gibt es Hinweise auf eine veränderte Dopamintransporteraktivität. Diese Auffälligkeiten könnten die Wirksamkeit antipsychotisch wirksamer Substanzen beeinflussen und letztlich zur Entwicklung von Behandlungsresistenz bei Psychosen beitragen [16].

### Merke

Eine pflanzenbetonte, ballaststoffreiche, wenig verarbeitete und vollwertige – naturnahe – Ernährung beeinflusst über Biomarker molekulare Mechanismen und das Mikrobiom kognitive Funktionen und mentale Gesundheit positiv. Ernährungsinterventionen sind eine wichtige Ergänzung herkömmlicher Therapien.

### Infobox 1 Mikrobiom

Das Mikrobiom – die Gesamtheit aller Mikroorganismen eines Wirts – und ihre jeweiligen Stoffwechselprodukte kommunizieren über eine Reihe biochemischer und funktioneller Verbindungen mit dem Wirt und beeinflussen so dessen Homöostase und Gesundheit. Insbesondere kommuniziert der Magen-Darm-Trakt über die Darm-Hirn-Achse mit dem Zentralnervensystem, um die Entwicklung und Aufrechterhaltung von Neuronen zu unterstützen, während sich eine Darmdysbiose wohl in neuropsychiatrischen Störungen manifestiert. Es gibt drei grundlegende Mechanismen, welche die Kommunikation zwischen dem Darm und dem Gehirn vermitteln: direkte neuronale Kommunikation, endokrine Signalübermittler und das Immunsystem. Zusammen bilden diese Systeme ein hochgradig integriertes molekulares Kommunikationsnetz, das systemische Ungleichgewichte mit der Entwicklung von Neurodegeneration verbindet, einschließlich der Insulinregulierung, des Fettstoffwechsels, der oxidativen Marker und der Immunsignalgebung [17, 18]. Das Gleichgewicht des Mikrobioms kann beispielsweise durch Süßstoffe [19], aber auch Konservierungs- und Zusatzstoffe wie Emulgatoren und besonders durch Alkoholkonsum in Dysbalance geraten. Auch legen Daten nahe, dass Veränderungen im Mikrobiom mit der Entstehung von Lebererkrankungen bei Alkoholabusus durch inflammatorische Prozesse in Zusammenhang stehen [20]. Auch Antibiotikagaben im Übermaß führen zu einer veränderten Zusammensetzung des Mikrobioms und wiederholte Einnahmen scheinen Antibiotikaresistenzen zu fördern [21].

## Gib es ein Suchtpotenzial bei verdichteten Kohlenhydraten?

Der Konsum von Zucker ist für die meisten Menschen schmackhaft und für den Moment wohltuend aufgrund eines starken Einflusses auf unser **Belohnungssystem**

**Abb. 2:**
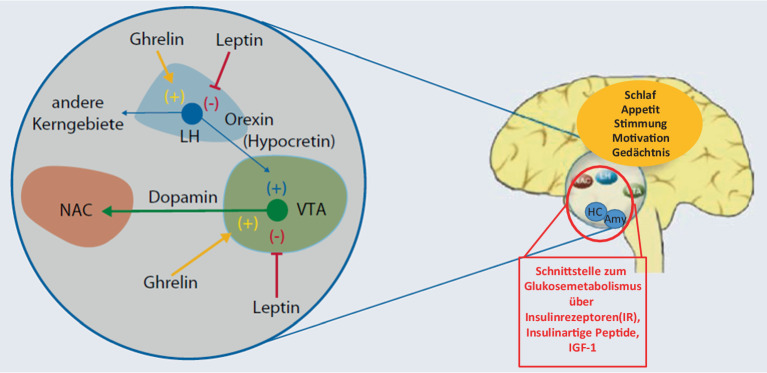
Appetit, Insulin, Stimmung und Kognition hängen zusammen: Appetitregulierende Peptide Leptin, Ghrelin und Orexin verknüpfen energiehomöostaseregulierende Systeme (lateraler Hypothalamus, *LH*) mit dem dopaminergen mesolimbischen System, mit Projektionen zum ventralen Tegmentum (*VTA*) und Nucleus accumbens (*NAC*). Zudem besteht eine enge Beziehung des Glukosemetabolismus zu stresssensiblen Kerngebieten der Emotionsregulation (*NAC*, Hippokampus [*HC*] und Amygdala [*Amy*]). *IGF1* „insulin-like growth factor 1“. (Mod. aus [13])

Belohnungssystem

(Abb. 2). Durch wiederholte Aufnahme von hochverdichteten Kohlenhydraten insbesondere in Kombination mit Fett (z. B. paniertes Schnitzel mit Pommes, Käsekuchen, Creme-Desserts [22, 23]) kommt es zur **Dopaminausschüttung**

Dopaminausschüttung

als Schlüsseltransmitter im Rewardsystem, letztlich aber auch dadurch zu Veränderungen der Dopaminrezeptoraktivität, was mit einer veränderten Belohnungsverarbeitung und Sensibilität einhergehen kann und das Verlangen nach Zucker/Fett steigert. Langfristig kann dies zu einer Desensibilisierung des dopaminergen Regelkreislaufs führen, bei der mehr Nahrung benötigt wird, um das gleiche Belohnungsgefühl zu erzielen. Diese neuroadaptiven Veränderungen können letztlich zu einer **Entkopplung des Essverhaltens**

Entkopplung des Essverhaltens

von den kalorischen Bedürfnissen führen, was zu wiederkehrendem Überessen („die ganze Tafel Schokolade auf einmal“) und im Verlauf mit einer Gewichtszunahme einhergeht – die Entwicklung eines metabolischen Syndroms (MetS) oder T2DM, aber auch die Zunahme inflammatorischer Prozesse sind die Folgen [24, 25].

Ob es tatsächlich eine Art **„Zuckersucht“**

„Zuckersucht“

gibt, wird in der Wissenschaft kontrovers diskutiert, sicher ist jedoch, dass beim Essen von Zucker besonders in Kombination mit **Fett**

Fett

überlappende Regionen im Belohnungssystem des Gehirns wie bei einem Konsum von Suchtstoffen aktiviert werden.

Eine Forschergruppe um Bartley Hoebel hat die Effekte erhöhten Zuckerkonsums genauer untersucht. In der Studie wurde Ratten zunächst die morgendliche Fütterung verwehrt und erst im Intervall Zugang zu einer Zuckerlösung gegeben. Nach Konsum konnte ein Steigen des Dopaminlevels im Bereich des **Nucleus accumbens**

Nucleus accumbens

beobachtet werden. Im Verlauf der Intervention nahmen sowohl die Dopaminausschüttung als auch die Dichte der **D2-Rezeptoren**

D2-Rezeptoren

ab, so wie es auch bei anderen Abhängigkeitserkrankungen beobachtet werden kann. Die Tiere zeigten Entzugssymptome und ängstliches Verhalten bei fehlender Zuckergabe, was seitens der Wissenschaftler als Hinweis auf ein mögliches Suchtpotenzial von Zucker gedeutet wird [26].

Die Auswirkungen von Dopamin im Gehirn sind dabei möglicherweise nicht auf hedonische Wege der Nahrungsbelohnung beschränkt. Zum Beispiel kann striatales Dopamin auch nachgeschaltete **Hypothalamuskerne**

Hypothalamuskerne

beeinflussen, die traditionell auch für die Kontrolle der homöostatischen Ernährung und die Regulierung des Körpergewichts verantwortlich gemacht werden, was letztendlich die Aufnahme von Nahrungsmitteln fördert, die Fettleibigkeit verursachen, und die Abwertung von Lebensmitteln, die nicht zu Fettleibigkeit führen. Es ist daher faszinierend zu spekulieren, dass die Zusammensetzung der Ernährung dazu beitragen kann, den homöostatischen „Sollwert“ des Körpergewichts durch Veränderungen des Dopamins im Gehirn zu verändern [23].

Dazu passend zeigte sich mittels PET(Positronenemissionstomographie)-Bildgebung beim Menschen, dass sowohl bei Fettleibigen als auch bei Abhängigkeitserkrankten die **Dopamin-D2-Rezeptordichte**

Dopamin-D2-Rezeptordichte

im Striatum im Vergleich zur Kontrollgruppe reduziert ist. Dieselbe Arbeitsgruppe um Wang et al. stellte überdies fest, dass die Verfügbarkeit von D2-Rezeptoren bei adipösen Probanden negativ mit dem BMI korreliert [27].

Daten aus der funktionellen Magnetresonanztomographie (fMRI) weisen darüber hinaus darauf hin, dass es bei visueller Präsentation hochkalorischer Nahrungsmittel zu einer Aktivierung mesolimbischer Areale kommt, die zum einen mit dem Belohnungssystem, zum anderen mit dem Erlernen von Gewohnheiten in Zusammenhang stehen [28].

### Merke

Eine Desensibilisierung des dopaminergen Regelkreislaufs durch übermäßigen Zucker- und Fettkonsum kann zu einem erhöhten Bedarf an Nahrung führen, um dasselbe Belohnungsgefühl zu erzielen.

Einschränkend ist hier zu erwähnen, dass bisher ein umfassendes Modell zur Beziehung von Dopamin (DA) und Fettleibigkeit fehlt. Insbesondere die Humandaten sind nach wie vor nicht eindeutig. Nichtlineare Veränderungen des allgemeinen DA-Tonus vom Normalen zu Übergewicht hin zu Adipositas sollten mitgedacht werden. So könnten **Veränderungen des dopaminergen Tonus**

Veränderungen des dopaminergen Tonus

mit zunehmender Adipositas eher als Störungen der dopaminergen Regulation denn als Defizite in der DA-Übertragung angesehen werden, vergleichbar mit z. B. Psychosen. Übergewicht und leichte Adipositas könnten mit einer Verringerung des dopaminergen Tonus und damit verbundenen übertriebenen phasischen DA-Reaktionen im Striatum einhergehen. Im Gegensatz dazu kann eine schwere Adipositas durch einen insgesamt erhöhten dopaminergen Tonus mit assoziiertem reduziertem striatalem DA-Burst gekennzeichnet sein. In diesem Bereich besteht noch Forschungsbedarf [29].

Weitere Daten weisen darauf hin, dass es einen Zusammenhang zwischen **Leptin**

Leptin

(Abb. 2) und der Aktivierung von Belohnungswegen im Gehirn als Reaktion auf Nahrungsmittelreize gibt. Leptin ist ein Hormon, das hauptsächlich von **Fettzellen**

Fettzellen

produziert wird und eine wichtige Rolle bei der Regulierung des Energiehaushalts des Körpers spielt. Leptin signalisiert vereinfacht gesagt dem Gehirn den aktuellen Fettgehalt im Körper, um Nahrungsaufnahme und Energieverbrauch zu steuern. Es beeinflusst somit Appetit und Stoffwechsel. Eine **Leptinresistenz**

Leptinresistenz,

bei welcher der Körper weniger sensibel auf Leptin reagiert, kann zu Überessen und Gewichtszunahme führen, was häufig bei Adipositas beobachtet wird [30]. Bei einer Untersuchung von adipösen und normalgewichtigen Personen wurde festgestellt, dass eine höhere Konzentration von Leptin im Blut mit einer gesteigerten Aktivierung des ventralen Striatums, einem wichtigen Teil des Belohnungssystems des Gehirns, korreliert. Dies deutet darauf hin, dass Leptin eine Rolle in der Modulation der Reaktion des Belohnungssystems auf Nahrungsreize spielt. Es wird vermutet, dass eine gestörte Rückkopplungsregulation der Belohnungsbahnen bei fettleibigen Personen zu suchtähnlichem Verhalten und der Unfähigkeit führt, die Nahrungsaufnahme an physiologische Bedürfnisse anzupassen [31].

### Merke

Leptin steuert Appetit, Nahrungsaufnahme, Energieverbrauch und reguliert den Energiehaushalt und Stoffwechsel. Leptinresistenz führt zu Überessen und Gewichtszunahme.

## Die Rolle von Insulin im Gehirn

Obwohl Insulin oft ausschließlich mit einer peripheren Wirkung im Körper in Verbindung gebracht wurde, haben mehrere Studien gezeigt, dass Insulin auch im Gehirn eine wichtige Rolle spielt, da es das **Sättigungsgefühl**

Sättigungsgefühl

über den Hypothalamus reguliert (Abb. 2) und Insulinrezeptoren (IR) u. a. auch in der Hippokampus-Amygdala-Region exprimiert werden. Diese Erkenntnisse belegen die Bedeutung von Insulin für lebenswichtige Funktionen des Gehirns, einschließlich Energieerhaltung, Stimmung und Gedächtnisbildung. Durch die Bindung an seinen Rezeptor aktiviert Insulin eine Signalübertragung, deren Defekt eine Rolle bei Funktionsstörungen des Gehirns spielt und zu neurodegenerativen Erkrankungen, insbesondere der **Alzheimer-Krankheit**

Alzheimer-Krankheit

(AD), führen könnte. Da die Insulinsignalübertragung für die synaptische Plastizität, das Gedächtnis und die Langzeitpotenzierung von wesentlicher Bedeutung ist, spielt der gestörte Insulinweg folglich auch eine Rolle bei der Entstehung von AD. Der Zusammenhang zwischen Insulinresistenz und AD wurde in Nagetiermodellen der AD, bei fettreicher Ernährung und in einem Modell für nichtmenschliche Primaten (NHP) nachgewiesen. In Übereinstimmung mit diesen Ergebnissen haben klinische Studien Hyperinsulinämie mit einem erhöhten Risiko kognitiver Dysfunktion in Verbindung gebracht und einen Zusammenhang zwischen peripherer und zentraler Insulinresistenz bei AD gezeigt [32, 33].

Die **systemische Insulinresistenz**

systemische Insulinresistenz

ist ein Syndrom, das mit verschiedenen Erkrankungen wie T2DM, Bluthochdruck und Adipositas einhergeht. Es gibt immer mehr Belege dafür, dass die Insulinresistenz das Bindeglied zwischen T2DM, Adipositas, nichtalkoholischer Steatohepatitis (NASH) und **Hirnfunktionsstörungen**

Hirnfunktionsstörungen

ist, die sich auf Intelligenz, Emotionsregulation, Kognition, Lernen wie Gedächtnis auswirken. Insbesondere haben epidemiologische Studien gezeigt, dass T2DM, Adipositas und andere prädiabetische Zustände der Insulinresistenz Risikofaktoren für die AD und verwandte Störungen sind [32, 34, 35, 36].

### Merke

Insulinrezeptoren im Gehirn beeinflussen Appetit und kognitive Funktionen. Die systemische Insulinresistenz stellt einen Risikofaktor für AD und neurodegenerative Erkrankungen dar.

## Kalorienrestriktion und das Gehirn

Grundsätzlich kann man zwischen einer langfristigen, einer intermediären Kalorienrestriktion und kurzfristigen Nulldiäten mit deutlichem Gewichtsverlust von > 10 % unterscheiden.

Eine **komplette Kalorienrestriktion**

komplette Kalorienrestriktion

wird medizinisch seit langem nicht mehr durchgeführt, sollte anamnestisch jedoch in der Praxis erfragt werden und auf mögliche Risiken in der Psychoedukation hingewiesen werden. Stattdessen haben sich **periodisches Fasten**

periodisches Fasten

(z. B. Intervallfasten), modifiziertes Fasten (z. B. Buchinger-Fasten) und die „fasting-mimicking diet“ (Scheinfasten mit bis zu 1000 Kcal/Tag) durchgesetzt. Für diese liegt eine umfangreiche klinische Evidenz für kardiovaskuläre Erkrankungen, T2DM sowie chronische Inflammation vor, auch wenn bisher gute Langzeitstudien zu Vor- und Nachteilen fehlen. Zur Vertiefung der verschiedenen Formen der Fastentherapie, erforschter Indikationsgebiete und auch Fastenhelfer sei auf die aktuelle Übersicht von Hanslian et al. verwiesen [37].

### Langfristig ist günstig

Lange ist bekannt, dass wenn die Nahrungsaufnahme von Organismen wie Hefezelle, Drosophila oder Nagetieren reduziert wird – besonders in der zweiten Lebenshälfte – diese länger leben als Organismen, die eine normale Ernährung beibehalten. Bei Nagetieren können sowohl eine diätetische Einschränkung als auch eine verminderte Aktivität von Nährstoffsensoren die Häufigkeit altersbedingter Funktionsverluste und Krankheiten, einschließlich Tumoren und Neurodegeneration, senken. Medizinisches Fasten kann daher den **Alterungsprozess**

Alterungsprozess

durch ähnliche Transduktionskaskaden verlangsamen, die während der Evolution speziesübergreifend konserviert wurden [38, 39].

Eine langfristige, **leichte Kalorienrestriktion**

leichte Kalorienrestriktion

(CR) bei erwachsenen NPH reduziert ebenfalls die Morbidität signifikant und erhöht das mediane Sterbealter. Affen [39], die im Alter von etwa 10 Jahren zunächst kalorienbeschränkt waren, wurden über 15 Jahre weiter beobachtet. Die mittlere **Lebenserwartung**

Lebenserwartung

dieser Affen nahm zu und überstieg die von ad libitum (AL) gefütterten Affen. Ähnliches konnte für Menschen in der CALERIE-Studie nachgewiesen werden. Eine zweijährige CR bei nicht übergewichtigen Menschen führte zu einer Reduktion von Gewicht, Taillenumfang und Gesamtfettmasse, was letztlich ebenfalls mit einem günstigen Einfluss auf die Lebenserwartung einhergeht [40].

Diese Erkenntnisse deuten darauf hin, dass ein Großteil der adipositasassoziierten Morbidität durch **anhaltende, leichte Kalorienzurückhaltung**

anhaltende Kalorienzurückhaltung

gemildert werden könnte. Darüber hinaus wird wahrscheinlich aufgrund der Vorbeugung von Fettleibigkeit auch die Entwicklung eines T2DM verhindert. Zu den Erkenntnissen gehören die Identifizierung von Veränderungen im **Glykogensyntheseweg**

Glykogensyntheseweg

und bei der Phosphorylierung der Glykogensynthase als Reaktion auf Insulin. Diese durch CR induzierte Vorbeugung von Morbidität erfordert keine übermäßige Magerkeit, sondern ist vorhanden, wenn das **Körperfett im Normbereich**

Körperfett im Normbereich

von 10–22 % liegt [41].

#### Merke

Langfristige, kontrollierte Kalorienrestriktion scheint Morbidität zu senken, Alterungsprozesse zu verlangsamen und die Lebensdauer zu erhöhen.

### Intermediäre Kalorienrestriktion

Ein wiederentdeckter Ansatz beim Menschen ist das intermittierende Fasten (IF), bei dem im Vergleich zu klassischer Kalorienrestriktion nur die **Zeit der Nahrungsaufnahme**

Zeit der Nahrungsaufnahme

und nicht die Anzahl der pro Tag erlaubten Kalorien begrenzt wird. Es gibt bereits eine Vielzahl von Belegen aus präklinischen und klinischen Studien, welche die positiven Auswirkungen des IF belegen. Es gibt wichtige molekulare Akteure, die während des IF im Gehirn verändert werden und an ihren positiven zentralen Wirkungen beteiligt sind. Darunter wurden Ketonkörper, BDNF („brain-derived neurotrophic factor“), GABA (γ-Aminobuttersäure), GH/IGF‑1 („growth hormone“/„insulin-like growth factor 1“), FGF21 („fibroblast growth factor 21“), Sirtuin‑3, mTOR und die Darmmikrobiota (siehe Infobox 2) identifiziert. Studien deuten darauf hin, dass IF mehrere molekulare und zelluläre Anpassungen in Neuronen hervorruft, die insgesamt die zelluläre Stressresistenz, **synaptische Plastizität**

synaptische Plastizität

und Neurogenese verbessern [42].

Das Fehlen von Leitlinien für die Anwendung des IF bei Patienten behindert jedoch bisher seine breite Anwendung in der klinischen Praxis und es sind weitere Studien erforderlich, um unser Wissen über die verschiedenen IF-Protokolle und die langfristigen Auswirkungen des IF auf den **Hirnstoffwechsel**

Hirnstoffwechsel

zu verbessern [43].

#### Merke

IF steigert die synaptische und zelluläre Neuroplastizität und hat positive Auswirkung auf mentale Gesundheit.

### Kurzfristige Nulldiät ist (zumindest bei Mäusen) langfristig eher ungünstig

Im Gegensatz dazu führt schon eine 10-tägige weitgehend komplette Kalorienrestriktion (CCR) mit Gewichtsreduktion um 10 % zu langfristigen Veränderungen im Ernährungsverhalten und in der Regulierung des Körpergewichts sowie auf der Ebene der Epigenetik in Nervenzellen des Gehirns. Nach CCR wurden Mäuse bezüglich Verhaltens- und Stoffwechsel untersucht. Der Transkriptionsfaktor **∆FosB**

∆FosB

wurde spezifisch im **Nucleus accumbens**

Nucleus accumbens

(NAc) mit viral vermitteltem Gentransfer gemessen und Verhaltens- und Stoffwechselstudien durchgeführt.

Es zeigte sich, dass die Akkumulation von ∆FosB in der NAc-Schale nach CCR bei Mäusen mit konsekutiv **erhöhter Fettaufnahme**

erhöhter Fettaufnahme

und einem reduzierten Energieverbrauch einhergeht. Darüber hinaus senkte die Überexpression von ∆FosB in dieser Region über einen orexinabhängigen Mechanismus den Energieverbrauch und förderte Übergewicht.

Diese Ergebnisse deuten darauf hin, dass der ∆FosB-Signalweg in NAc langanhaltende adaptive Reaktionen auf CCR vermittelt, die mit **Gewichtszunahme**

Gewichtszunahme

assoziiert ist [44].

#### Merke

Eine komplette Kalorienrestriktion über längere Zeit hat nichts mit medizinisch begleitetem Fasten zu tun. Biologisch ist CCR ein erheblicher Stressfaktor und fördert eher Übergewicht durch langanhaltende adaptive Reaktionen im Gehirn.

### Gewichtsverlust bei Menschen mit Adipositas und das Gehirn

Nährstoffsignale von Fett und Kohlenhydraten an das Gehirn regulieren das Essverhalten bei Nagetieren. Reaktionen des Gehirns auf diese Signale werden mit pathologischem Fressverhalten und Fettleibigkeit in Verbindung gebracht.

Um dies am Menschen zu überprüfen, erfolgte eine einfach verblindete, randomisierte, kontrollierte, ganz aktuelle **Crossover-Studie**

Crossover-Studie

an 30 Menschen mit einem normalen Körpergewicht (Frauen *N* = 12, Männer *N* = 18) und 30 Menschen mit Fettleibigkeit (Frauen *N* = 18, Männer *N* = 12, BMI<35). Die Wirkung intragastrischer Glukose‑, Lipid- und Wasserinfusionen (nichtkalorische isovolumetrische Kontrolle) auf die primären Endpunkte „zerebrale neuronale Aktivität“ und „striatale Dopaminfreisetzung“ sowie auf die sekundären Endpunkte „Plasmahormone und Glukose“, „Hunger-Scores“ und „Kalorienaufnahme“ wurden untersucht. Um zu prüfen, ob Reaktionen bei Teilnehmern mit Adipositas bei diätbedingtem Gewichtsverlust teilweise reversibel sind, wurde die Bildgebung nach einem diätinduzierten Gewichtsverlust von 10 % wiederholt.

Es zeigte sich, dass intragastrische Glukose- und Lipidinfusionen – unabhängig von Geruch, Geschmack oder Präferenz – nährstoffspezifische zerebrale neuronale Aktivität mit Reduktion **striataler Dopaminfreisetzung**

striatale Dopaminfreisetzung

bei schlanken Teilnehmern induzieren. Im Gegensatz dazu haben Teilnehmer mit Adipositas im fMRI eine gegenläufige Gehirnreaktion im dopaminergen NAc auf Nährstoffe nach der Einnahme und in der Peripherie eine erhöhte Insulinantwort und einen verminderten GLP-1-Anstieg. Wichtig ist, dass die veränderten neuronalen Reaktionen nach einer diätbedingten Gewichtsabnahme nicht wiederhergestellt werden. Diese anhaltend veränderten neuronalen Reaktionen auf Ernährungssignale können zu übermäßigem Essen und Fettleibigkeit beitragen. **Veränderte Nährstoffsignalverarbeitung**

Veränderte Nährstoffsignalverarbeitung

nach signifikanter Gewichtsabnahme kann zum Teil die hohe Rate der Gewichtszunahme nach erfolgreicher Gewichtsabnahme erklären [45].

#### Infobox 2 Resistente Stärke: Ballaststoff für den Darm

Resistente Stärke findet sich nicht nur in Samen und Getreidekörnern (Typ 1) sowie Bananen (Typ 2), sondern entsteht auch durch das Abkühlen gekochter stärkehaltiger Nahrungsmittel wie Kartoffeln, Reis und Nudeln (Typ 3). Für den Darm wird so aus beliebten Kohlenhydraten ein Ballaststoff. In einer aktuellen randomisierten, placebokontrollierten Crossover-Design-Studie an Teilnehmern mit Übergewicht oder Adipositas wurde untersucht, ob resistente Stärke (RS) als Nahrungsergänzungsmittel die adipositasbedingten Endpunkte beeinflusst. Hier zeigte sich, dass eine 8‑wöchige RS-Supplementierung (40 g/Tag) dazu beitragen kann, eine Gewichtsabnahme (Mittelwert −2,8 kg) zu erreichen und die Insulinresistenz bei Personen mit Übergewicht zu verbessern. Die Vorteile von RS sind mit Veränderungen der Zusammensetzung der Darmmikrobiota verbunden. Die Supplementierung mit *Bifidobacterium adolescentis*, einer Spezies, die bei den Studienteilnehmern mit der Reduktion von Fettleibigkeit assoziiert ist, schützt männliche Mäuse vor ernährungsbedingter Fettleibigkeit. Mechanistisch verändern die RS-induzierten Veränderungen der Darmmikrobiota das Gallensäureprofil, reduzieren Entzündungen durch Wiederherstellung der Darmbarriere und hemmen die Lipidaufnahme. Es zeigte sich, dass RS die Gewichtsabnahme zumindest teilweise durch *B. adolescentis* erleichtern kann und dass die Darmmikrobiota für die Wirkung von RS essenziell ist [46].

## Schlaf, Ernährung und Psyche

Regelrechter Schlaf spielt ebenfalls eine zentrale Rolle in der Regulierung des Stoffwechsels und beeinflusst direkt Faktoren, die sowohl mit der Entwicklung von Übergewicht, aber auch mit psychischer Gesundheit in Verbindung stehen. So ist lange die Interaktion von **Orexin-Neuronen**

Orexin-Neuronen

und dem monoaminergen System bekannt (Abb. 2). Eine **unzureichende Schlafdauer**

unzureichende Schlafdauer

und -qualität sind mit einer erhöhten Prävalenz von Adipositas und der Entwicklung eines T2DM verbunden [47]. So legen Daten nahe, dass bereits eine Nacht mit verkürzter Schlafdauer durch **Methylierungsprozesse**

Methylierungsprozesse

die Glykolyse in Muskelzellen hemmt und im Gegensatz dazu die Fettspeicherung in Adipozyten steigert. Auch erhöhte Kortisolspiegel und damit verbundene Entzündungsreaktionen scheinen eine Rolle zu spielen [48]. Schlafmangel beeinflusst darüber hinaus **hormonelle Prozesse**

hormonelle Prozesse,

die für die Regulation von Hunger und Sättigung verantwortlich sind. Studien zeigen, dass Schlafentzug zu einer Verringerung des Hormons Leptin führt und damit das Sättigungsgefühl vermindert. Überdies kommt es zu einem Anstieg des Hormons Ghrelin, das wiederum den Appetit stimuliert. Letztlich können diese Veränderungen mit einem gesteigerten Hungergefühl und einer erhöhten Nahrungsaufnahme einhergehen ([49]; siehe auch Abb. 2).

In einer weiteren Studie, die den Zusammenhang zwischen Schlafentzug und der Gehirnreaktion auf visuelle Nahrungsmittelstimuli untersucht hat, zeigte sich, dass akuter Schlafmangel die **Verarbeitung visueller Reize**

Verarbeitung visueller Reize,

die Nahrungsmittelkonsum anregen, im Gehirn verstärkt. Insbesondere wurde eine Aktivierung im Bereich des rechten anterioren zingulären Kortex nach Präsentation von Fotos mit Nahrungsmitteln deutlich. Auch das **Hungergefühl**

Hungergefühl

wurde von den schlafdeprivierten StudienteilnehmerInnen am Morgen als erhöht beschrieben, obschon die Plasmaglukosespiegel im Vergleich zur Kontrollgruppe unverändert waren [50].

Schlafstörungen sind darüber hinaus mit **psychischen Erkrankungen**

psychischen Erkrankungen

aller Art vergesellschaftet und beeinträchtigen als eigene Krankheitsentität stark die Lebensqualität. Durch eine damit einhergehende **Verminderung von Alltagsaktivität**

Verminderung von Aktivität

kommt es zu einem geringeren Energieverbrauch, welcher nicht zuletzt mit einer Gewichtszunahme einhergehen kann. **Schlafapnoe**

Schlafapnoe

und damit verbundene weitere Beeinträchtigungen der Schlafqualität sind differentialdiagnostisch zu bedenken. Schlafstörungen sollten im psychiatrischen Kontext als eigene Krankheitsentität gesehen werden, somatische Ursachen ausgeschlossen und leitliniengerecht zunächst psychotherapeutisch behandelt werden. Dementsprechende Programme wie beispielsweise SleepExpert [51] oder digitale Gesundheitsanwendungen (DiGA) wie Somnio können hierbei unterstützen. Mit einem **Orexinantagonisten**

Orexinantagonisten

steht in Ergänzung ein neuer medikamentöser therapeutischer Ansatz zur Behandlung von chronischer Insomnie (International Statistical Classification of Diseases and Related Health Problems 11, ICD-11) zur Verfügung [52].

Durch das **glymphatische System**

glymphatische System

werden zelluläre Abfallstoffe im zentralen Nervensystem abtransportiert. Der Prozess existiert in Wirbeltieren und ist fast vorwiegend während des Schlafes aktiv [53]. Störungen des Nachtschlafs werden mit einer verminderten Funktion dieses **„Reinigungsprozesses“**

„Reinigungsprozesses“

in Verbindung gebracht und damit als mitursächlich für neurodegenerative Erkrankungen, welche mit zerebralen Ablagerungen wie beispielsweise β‑Amyloid bei der AD einhergehen, diskutiert [54].

Abschließend sollte erwähnt werden, dass die Empfehlung zur Verlängerung der Schlafdauer auf 8,5 h pro Nacht z. B. durch Reduktion der Bildschirmzeit bei adipösen Erwachsenen zu einer verringerten Energiezufuhr und folglich zu einer Gewichtsabnahme führte [55].

### Merke

Schlafmangel und eine chronische Insomnie erhöhen durch eine Beeinträchtigung der Appetitregulation und Veränderung der Gehirnaktivität das Risiko für Adipositas und ein metabolisches Syndrom. Schlafstörungen begünstigen psychische Störungen, neurodegenerative Erkrankungen und sollten leitliniengerecht therapiert werden. Bereits die Verlängerung der Schlafdauer auf 8,5 h pro Nacht kann günstige Effekte auf Übergewichtigkeit mit sich bringen.

## Brain Food: Welche Ernährung nutzt dem Gehirn?

In den letzten Jahrzehnten wurden die traditionellen, regionalen Ernährungsgewohnheiten durch eine stärker **globalisierte Ernährung**

globalisierte Ernährung

ersetzt, die reich an **prozessierten Nahrungsmitteln**

prozessierten Nahrungsmitteln

mit gesättigten Fettsäuren und Einfachzucker ist, wohingegen der Anteil an günstigen Polyphenolen, Ballaststoffen und kurzkettigen Fettsäuren abgenommen hat. Es gibt zahlreiche Hinweise dafür, dass diese Ernährungsfaktoren zu einer Beeinträchtigung der kognitiven Funktionen und Neuroplastizität beitragen und das Auftreten von Stoffwechselkrankheiten wie Adipositas und T2DM erhöhen. Wie diese Nährstoffe die synaptische Funktion und die Neuroplastizität modulieren, ist jedoch wenig untersucht. Die Auswirkungen einer westlichen, einer mediterranen, einer ketogenen oder einer paläolithischen Ernährung auf die Kognition und auf Korrelationen mit synaptischen Veränderungen wurden hauptsächlich in kleinen Tierstudien untersucht (Übersicht siehe Tab. 1). Gedächtnis- und Lerndefizite, die durch fett- und zuckerreiche Ernährung vermittelt werden, sind – selbst über kurze Expositionszeiten – wohl mit einer verringerten **Arborisierung der Dendriten**

Arborisierung der Dendriten,

einem vergrößerten synaptischen Spalt, einer verengten **postsynaptischen Zone**

postsynaptischen Zone

und einer verringerten aktivitätsabhängigen synaptischen **Plastizität im Hippokampus**

Plastizität im Hippokampus

verbunden. Es wurde gezeigt, dass diese Veränderungen mit einer Deregulierung der ionotropen Glutamatrezeptoren vom AMPA(Aminomethylphosphonsäure)-Typ korrelieren, die für die Neuroplastizität entscheidend sind [56].

**Tab. 1:** Beschreibung der unterschiedlichen Ernährungsformen. (Nach [56, 57, 58, 59])

Ernährungsform	Zusammensetzung	Effekte auf Kognition, Plastizität und Stoffwechsel	Bewertung
Westlich	Hoher Anteil an tierischen Lebensmitteln, gesättigten Fetten und Einfachzucker	*Ungünstige Effekte* auf Adipositas, T2DM, MetS, Lern- und Gedächtnisfunktionen, besonders bei Älteren Induktion von Neuroinflammation und Amyloidanstieg	Relativ gute Datenlage für ungünstige Effekte
Fettreich (Hoch-Fett)	35–60 % Kalorien aus unterschiedlichen Fetten	Bei gesättigten Fettsäuren: Hinweise für synaptische Dysfunktion und Gedächtniseinbußen. *Hoher Anteil ungesättigter Fettsäuren wirken dem entgegen*	Studienlage im Hinblick auf Methodik derzeit schlecht
Hoch-Zucker	Hoher Anteil an verdichteten Kohlenhydraten und Fertigprodukten	*Ungünstige Effekte* auf Adipositas, T2DM, MetS, kognitive Funktionen, Mikrobiom, Inflammation. Fruktose im Vergleich ungünstiger als Saccharose, besonders bei Männern und Älteren	Relativ gute Datenlage für ungünstige Effekte
Ketogen	Reich an (ungesättigten) Fetten und arm an Kohlenhydraten zur Induktion der Bildung von Ketonkörpern	Hinweise: kognitive Beeinträchtigungen wirksam reduziert, reduzierte GLUT-1-Konzentration, reduziert Insulinresistenz. *Günstige Effekte* auf T2DM, AD und juvenile Epilepsie [60]	Gute Daten bei T2DM, AD, juveniler Epilepsie, keine sicheren Belege für Effekte bei Gesunden, ggf. Erhöhung des kardiovaskulären Risikos [61] Weitere Studien erforderlich
Mediterran, MIND für Senioren	Reich an Gemüse, Obst, Ballaststoffen, Vollkornprodukten, Olivenöl, Fisch, wenig rotes Fleisch und raffinierte Zucker	Hinweise: verbesserte Lern- und Gedächtnisfunktionen *Günstige Effekte* auf Gewicht, Diabetes und MetS, Depression [62, 63]	Gute Hinweise, geringe Effekte, Studienlage muss aber verbessert werden
Paleolithisch	20–30 % Eiweiß, 30–34 % Kohlenhydrate, 30–40 % Fett: Obst, Gemüse, Nüsse, Fisch, Eier, mageres, unverarbeitetes Fleisch, wenig Getreide, Zerealien, Hülsenfrüchte, vollkommener Verzicht auf verarbeitete Nahrungsmittel	Hinweise: bei Übergewicht eher *günstige* Effekte auf Kognition, Lern- und Gedächtnisfunktion Serum-BDNF steigt *Wenig ungünstige Effekte* auf Gewicht, Diabetes und MetS	Studienlage sehr begrenzt, keine eindeutigen Schlussfolgerungen auf Plastizität und Kognition

*AD* Alzheimer-Demenz, *BDNF* „brain-derived neurotrophic factor“, *MetS* metabolisches Syndrom, *T2DM* Diabetes mellitus Typ 2,

Wie oben schon beschrieben, finden sich über diese neuronalen Dysfunktionen Assoziationen mit verschiedenen, stressassoziierten psychischen Störungen, insbesondere affektiven Störungen und mit begleitenden kognitiven Defiziten.

Auch einzelnen Lebensmittel wird nachgesagt, sie seien gut für das Gehirn. Aussagekräftige Studien zur Wirksamkeit fehlen jedoch, oft basieren die Daten in diesem Themenfeld auf retrospektiven Selbstberichten im Querschnitt mit Verzerrungseffekten. Hinweise aus den wenigen randomisierten, kontrollierten Studien gibt es für **Omega-3-Fettsäuren**

Omega-3-Fettsäuren,

B‑Vitamine und bestimmte **Pflanzenfarbstoffe**

Pflanzenfarbstoffe

aus Beeren. Das Gehirn profitiert aber weniger von einer isolierten Gabe dieser Lebensmittel, als vielmehr von einer abwechslungsreichen, naturnahen, jahreszeitlich und örtlich angepassten Kost im Sinne eines **„Langstreckenlaufs“**

„Langstreckenlaufs“.

Die Konsequenzen unserer ungünstigen Gewohnheiten machen sich also erst viel später bemerkbar [64, 65, 66].

Zusammenfassend spricht viel dafür, dass die Prämisse, dass eine hohe Fettaufnahme Fettleibigkeit, Diabetes, Herzerkrankungen und möglicherweise Krebs verursacht, heute so nicht mehr haltbar ist. Gute Befunde gibt es für die nachteiligen metabolischen Auswirkungen hochverarbeiteter Nahrungsmittel und die günstigen Effekte kohlenhydratarmer und ketogener Diäten mit hohem **ungesättigtem Fettgehalt**

ungesättigtem Fettgehalt

und reichlich **Ballaststoffen**

Ballaststoffen,

wozu auch resistente Stärke zählt (siehe Infobox 3). Dabei hat die relative Menge an Nahrungsfett und Kohlenhydraten wohl weniger Relevanz für die Gesundheit. Stattdessen sollte der Fokus daraufgelegt werden, welche bestimmten Fett- oder Kohlenhydratquellen konsumiert werden – auch zu welcher Tageszeit – und wie vielfältig und naturnah die Ernährungsgewohnheit insgesamt über die Lebenszeit ist [59].

### Merke

„Brain Food“ unterstützt neuronale Funktionen. Im Gegensatz zu westlichen Ernährungsformen mit vielen tierischen Produkten scheinen mediterrane Ernährungsformen mit hohen, pflanzenbetonten kurzkettigen Fettanteilen und pflanzlichen Produkten (auch Obst [67]) das Demenzrisiko zu senken [68] und sich günstig auf depressive Störungen auszuwirken [62, 63].

### Infobox 3 Darm, Stress, Vagus und Probiotika

Funktionelle Darmerkrankungen und stressassoziierte psychische Störungen wie Depression und Angst scheinen pathophysiologisch enge Verwandtschaften aufzuweisen [69]. Pathogene Bakterien führen im Nagetiermodell zu Angstsymptomen vermittelt über GABAerge vagale Afferenzen [70]. Umgekehrt gibt es immer mehr Hinweise, dass Probiotika günstig Einfluss nehmen können auf psychische Symptome wie sie beispielsweise bei chronischer Fatigue auftreten [71]. Experimentelle Daten im Tiermodell legen nahe, dass die regelmäßige Einnahme von beispielsweise* Lactobazillus rhamnosus,* ein probiotisches Milchsäurebakterium, zu Veränderungen GABAerger mRNA-Expression im Gehirn und damit zu einer Einflussnahme auf die Neurotransmission und auf das Verhalten (ängstlich/depressive Verhaltensweisen) führen könnte. Vagotomierte Mäuse zeigten diese Veränderungen wiederum nicht, was die Hypothese eines vagalen Transmissionsweges unterstreicht. Ein perspektivischer Einsatz von Probiotika bei stressassoziierten Erkrankungen erscheint denkbar [72].

## Pharmakologische Unterstützung bei Adipositas und Diabetes

Seit vielen Jahren wird versucht, nicht nur durch Ernährung, Bewegung und Schlaf, sondern auch pharmakologisch der „Adipositas-Pandemie“ zu begegnen. Die erfolgreichsten diesbezüglichen „Kandidaten“ kommen aus der Behandlung des Typ-2-Diabetes. Beispielhaft werden im Folgenden zwei Vertreter vorgestellt.

**Metformin**

Metformin,

ein Antihyperglykämikum der ersten Wahl, ist ein 5′-Adenosinmonophosphat(AMP)-aktivierter Proteinkinase(AMPK)-Aktivator, der zusätzlich einerseits einen **gewichtsreduzierenden Effekt**

gewichtsreduzierenden Effekt

hat [73] und gleichzeitig einhergeht mit **entzündungshemmenden Effekten**

entzündungshemmenden Effekten,

Verbesserung des Gedächtnisses und verlängerter Lebenserwartung. Darüber hinaus wurde kürzlich nachgewiesen, dass Metformin die Immunreaktivität von Synaptophysin, Sirtuin‑1, AMPK und „brain-derived neuronal factor“ erhöht, die wesentliche Marker der Neuroplastizität sind [74].

„Fettleibigkeit hat ihren Gegenspieler gefunden“ überschreibt das Wissenschaftsjournal *Science* seine ganz aktuelle Entscheidung, **GLP-1-Agonisten**

GLP-1-Agonisten

den Titel „Breakthrough of the Year 2023“ zu verleihen.

In den frühen 1980er-Jahren entdeckten Forschende, die sich mit Diabetes und der Blutzuckerregulation beschäftigten, im Darm das Inkretinhormon **„glucagon-like peptide-1“**

„glucagon-like peptide-1“

(GLP-1). Bereits in den 1990er-Jahren zeigten dann Experimente mit Ratten, dass die Tiere weniger Nahrung aufnahmen, wenn man ihnen GLP‑1 ins Gehirn injiziert hatte. In kleineren Studien konnte der **appetithemmende Effekt**

appetithemmende Effekt

auch bei gesunden jungen Männern gezeigt werden, denen man GLP‑1 intravenös verabreicht hatte.

In der Würdigung der großen Forschungsanstrengungen der GLP-1-Agonisten als Durchbruch des Jahres erinnert die Wissenschaftsjournalistin Jennifer Couzin-Frankel daran, dass mit den GLP-1-Agonisten erstmals Wirkstoffe zur Verfügung stehen, die das Ende einer unrühmlichen Vergangenheit pharmakologischer Verzweiflungstaten in Bezug auf Appetitzügler einläuten könnten und die sich auch günstig auf Risiken wie z. B. Herzinfarkt auswirken.

Vor übertriebenem Optimismus warnt jedoch in einem begleitenden Editorial der Chefredakteur von *Science*. Denn so vielversprechend die GLP-1-Agonisten auch sein mögen, so haben sie bisher mehr Fragen aufgeworfen als beantwortet. Zu den Problemen zählt er die **Kosten**

Kosten

und die schlechte **Verfügbarkeit**

Verfügbarkeit

der Arzneimittel. Zudem zeigt sich immer deutlicher, dass die therapeutischen Effekte sofort nachlassen, wenn die Wirkstoffe abgesetzt werden. Damit drängen sich Fragen nach den **Langzeiteffekten**

Langzeiteffekten

hinsichtlich Wirksamkeit und Verträglichkeit und auch begleitender, langfristiger Umstellung der Ernährungsgewohnheiten auf.

Aber eines haben GLP-1-Agonisten bereits jetzt gezeigt: Sie räumen mit dem diskriminierenden Narrativ auf, wonach Fettleibigkeit das Ergebnis einer mangelnden Willenskraft ist. Für ein krankhaftes Übergewicht sind u. a. genetische Aspekte, biochemische Prozesse, Verfügbarkeit hochverdichteter Nahrungsmittel, wiederholte, unbegleitete (Null‑)Diäten, aber nicht „geistige Beschränktheit“ verantwortlich. Diese Medikamente unterbrechen das übertriebene Verlangen nach Nahrungsaufnahme und stoppen einen (auch neuronal) aus der Kontrolle geratenen Appetit. Und allein diese Tatsache kann als ein Durchbruch gewertet werden – und verdeutlicht einmal wieder die Bedeutung des Gehirns [75, 76].

### Merke

Metformin und besonders GLP-1-Agonisten sind eine aussichtsreiche wirksame pharmakologische Unterstützung in der Adipositastherapie, verringern unkontrollierten Appetit, übermäßige Nahrungsaufnahme und wirken antiinflammatorisch.

## Fazit für die Praxis

- Ernährung, Stoffwechsel, Inflammation und Schlafqualität sind entscheidend für die Prävention und Therapie psychischer Störungen sowie neurodegenerativer Erkrankungen.
- Der Fokus sollte nicht nur auf der reinen Symptombehandlung liegen, sondern auf individuell und langfristig angepassten Ernährungsplänen und der Förderung eines gesunden Schlafes. Medizinisch begleitetes Fasten kann dabei auch ein Einstieg für bessere mentale Gesundheit sein.
- Interdisziplinäre Aufklärung über Präventionsmöglichkeiten durch Ernährungsgewohnheiten und Aspekte der Selbstwirksamkeit sind notwendig, um eine bestmögliche Behandlung für PatientInnen zu erreichen.
- Hinsichtlich medikamentöser Behandlungsaspekte der Adipositas und diätetischer, ergänzender Therapieoptionen besteht der Bedarf weiterer Forschung.
- Gute Kenntnisse in der Ernährungsmedizin sollten heute schon zur Psychiatrie und Psychotherapie gehören.

## CME-Fragebogen

Was postuliert das traditionelle Energiebilanzmodell als Hauptursache für Adipositas und Stoffwechselstörungen?

Genetische Faktoren reduzieren Energieverbrauch

Ungleichgewicht zwischen Kalorienaufnahme und -verbrauch

Mangel an körperlicher Aktivität allein

Qualität der aufgenommenen Nahrung

Stress und psychische Erkrankungen

Welcher der folgenden Aspekte wird durch Schlafmangel begünstigt?

Unterstützung einer gewünschten Gewichtsreduktion

Verbesserung der kognitiven Leistungsfähigkeit

Verminderung des Appetits durch Sensibilisierung von Belohnungsarealen

Erhöhtes Risiko der Entwicklung eines Diabetes

Vermindertes Verlangen nach zuckerhaltigen Nahrungsmitteln

Was ist ein Hauptmerkmal des Kohlenhydrat-Insulin-Modells?

Betonung der Kalorienanzahl in der Nahrung

Fokus auf den Verzehr hochverarbeiteter Kohlenhydrate und deren hormonelle Reaktionen

Verzicht auf alle Kohlenhydrate in der Ernährung

Kein Einfluss auf die Fettlagerung im Körper

Unabhängigkeit von der Nahrungsqualität

Wie beeinflusst das sog. „Brain Food“ die Gehirngesundheit?

Indem es den Konsum von Fast Food fördert

Durch den Verzicht auf jegliche Nahrungsaufnahme

Mit einer Erhöhung des Konsums bestimmter Nahrungsergänzungsmittel

Durch die Bereitstellung spezifischer Nährstoffe, die kognitive Funktionen und Gehirngesundheit unterstützen

Durch Veränderung der Fastenintervalle

Welche Wirkung hat intermittierender metabolischer Wechsel auf das Gehirn?

Verringerung der Neuroplastizität

Verschlechterung der kognitiven Funktionen

Steigerung der Neuroplastizität und Förderung der Gehirngesundheit

Hat nur geringe Auswirkungen auf das Gehirn selbst

Erhöht das Risiko für neurodegenerative Erkrankungen

Was ist wohl eine relevante Folge der Desensibilisierung des dopaminergen Regelkreislaufs durch übermäßigen Zuckerkonsum in Kombination mit Fett?

Erhöhter Bedarf an Nahrung zur Erzielung desselben Belohnungsgefühls

Verringerung des Appetits

Unverändertes Essverhalten

Abnahme des Körpergewichts

Verbesserung der kognitiven Leistungsfähigkeit

Welche Rolle spielen Insulinrezeptoren im Gehirn?

Sie haben keinen Einfluss auf die Gehirnfunktion.

Sie regulieren ausschließlich den Blutzuckerspiegel.

Sie beeinflussen Appetit, Reproduktionsfunktion und kognitive Funktionen.

Sie sind nur für die Kontrolle der Körpertemperatur verantwortlich.

Sie fördern die Fettlagerung im Gehirn.

Wie beeinflusst Schlafmangel das Risiko für Adipositas und metabolisches Syndrom?

Er führt zu einem geringeren Risiko für Adipositas.

Er hat keinen Einfluss auf das metabolische Syndrom.

Er verstärkt das Verlangen nach gesunder Ernährung.

Er erhöht das Risiko durch Beeinträchtigung der Appetitregulation und Veränderung der Gehirnaktivität.

Er verbessert die Insulinsensitivität.

Welche Funktion hat Leptin im Körper?

Es erhöht das Hungergefühl.

Es spielt keine Rolle im Energiestoffwechsel.

Es signalisiert Sättigung und hilft, die Energiebalance zu regulieren.

Es stimuliert den Appetit.

Es ist ausschließlich für den Kohlenhydratstoffwechsel verantwortlich.

Welche Auswirkung hat eine ketogene Diät auf die Gehirngesundheit?

Sie führt zu einer Verschlechterung der kognitiven Funktionen.

Sie hat keinen Einfluss auf die Gehirngesundheit.

Sie erhöht das Risiko für neurodegenerative Erkrankungen.

Sie fördert die Neuroplastizität und kann die Gehirngesundheit positiv beeinflussen.

Sie führt zu einem schnellen Verlust an Gehirnzellen.

## Abkürzungen

AD: Alzheimer-Erkrankung
BMI: Body-Mass-Index
CCR: Komplette Kalorienrestriktion
CIM: Kohlenhydrat-Insulin-Modell
CR: Kalorienrestriktion
fMRI: Funktionelle Magnetresonanztomographie
GABA: Gamma-Aminobuttersäure
GLP‑1: „Glucagon-like peptide 1“
IF: Intermittierendes Fasten
IR: Insulinrezeptor
MetS: Metabolisches Syndrom
NAc: Nucleus accumbens
NASH: Nichtalkoholische Steatohepatitis
NHP: Nichtmenschliche Primaten
T2DM: Diabetes mellitus Typ 2
